# Delayed Initial Radioiodine Adjuvant Therapy Does Affect Biochemical Response in Intermediate- to High-Risk Differentiated Thyroid Cancer

**DOI:** 10.3389/fendo.2021.743310

**Published:** 2021-11-09

**Authors:** Feng Yu, Xue Li, Yanhui Ji, Jian Tan, Guizhi Zhang, Peng Wang, Yajing He, Renfei Wang

**Affiliations:** ^1^ Department of Nuclear Medicine, Tianjin Medical University General Hospital, Tianjin, China; ^2^ Department of Nuclear Medicine, Shanghai Jiao Tong University Affiliated Sixth People’s Hospital, Shanghai, China

**Keywords:** Differentiated thyroid carcinoma (DTC), Radioiodine therapy, timing, therapy response, prognosis

## Abstract

**Background:**

There are no definite recommendations on the optimal time of initiating radioactive iodine (RAI) therapy for differentiated thyroid cancer (DTC) patients in current relevant guidelines. This study aimed to investigate the relationship between the timing of initiating radioiodine adjuvant therapy (RAT) and the clinical outcomes based on dynamic follow-ups and assessments in intermediate- to high-risk DTC patients.

**Methods:**

A total of 206 patients with intermediate- to high-risk DTC receiving RAT of 150 mCi were retrospectively reviewed. According to the time interval (TI: between thyroidectomy and initial RAT), the patients were divided into 2 groups: Group 1: TI < 3 months (n=148), and Group 2: TI ≥ 3 months (n=58). The RAT therapy response was evaluated as excellent response (ER), indeterminate response (IDR), biochemical incomplete response (BIR), structural incomplete response (SIR). The univariate and multivariate analyses were conducted to screen out factors associated with incomplete response (IR= BIR+SIR). Finally, the prognostic nomogram was used to explain IR rates as a valuable tool in clinical practice.

**Results:**

Response to initial RAT was significantly different between 2 groups during dynamic follow-ups (all P<0.05). Group 2 had significantly lower ER rates (37.9 *vs* 63.5, 52.0 *vs* 73.9, 64.4 *vs* 80.3, all *P*<0.05, respectively) and higher IR rates (39.7 *vs* 14.9, 36.0 *vs* 9.7, 12.2 *vs* 3.9, all *P*<0.05, respectively) than group 1 during dynamic follow-ups. By univariate and multivariate analyses, prolonged TI (HR: 6.67, 95%CI: 2.241-19.857, *P*=0.001), soft tissue invasion (HR: 7.35, 95%CI: 1.624-33.296, *P*=0.010), higher sTg (HR: 7.21, 95%CI: 1.991-26.075, *P*=0.003) were manifested to be independent risk factors for IR. The nomogram showed that soft tissue invasion, sTg, and TI were the top 3 contributors to the IR.

**Conclusions:**

Early RAT is associated with greater biochemical response but has no impact on SIR. Delayed initial RAT (≥3 months after thyroidectomy) related to IR in intermediate- to high-risk DTC.

## Introduction

The therapy methods of differentiated thyroid cancer (DTC) mainly include surgery, radioactive iodine (RAI) therapy, and thyrotropin (TSH) suppression therapy (1). Among them, postoperative selectivity RAI therapy is an essential supplement after thyroidectomy, playing a critical role in the purge of potential residual thyroid cancer and the therapy of distant metastases (2). According to the different purposes of RAI therapy, it mainly includes radioiodine remnant ablation (RRA), radioiodine adjuvant therapy (RAT) and radioiodine therapy of known disease (RTKD).

The timing of initiating the first post-thyroidectomy RAI therapy does matter for planning therapy when patients require this therapeutic modality (3–5). It varies around the world and may be affected by many factors, such as personal condition, social environment, and disease-related condition. To date, however, the optimal time of initiating RAI therapy remains not clear and there are no definite recommendations in current relevant guidelines (1, 6). Several studies have noted this tissue with different conclusions. Li et al (3) reported that delayed initial RRA (≥3 months after thyroidectomy) related to incomplete response (IR) in low- to intermediate-risk DTC. On the other hand, Kim et al (7) showed that delaying the first RTKD until 6 months after total thyroidectomy had no impact on restaging, recurrence and mortality in intermediate‐/high‐risk PTC. However, previous studies usually enrolled patients with different disease status, and they received a variable dosage of RAI for a different purpose, consequently, the results may be incomparable between studies.

With the re-recognition of the clinical value of RAI therapy, the number of DTC patients with higher serum stimulated thyroglobulin (sTg) level or poor clinicopathologic features who received the first RAT gradually increases, while the number of DTC patients who received RRA gradually decreases. Moreover, most previous studies observed clinical outcomes based on disease recurrence and mortality, not the new therapy response system proposed by the American Thyroid Association (ATA) guideline (version 2015) that divided clinical outcomes into four scenarios, including excellent response (ER), indeterminate response (IDR), biochemical incomplete response (BIR), structural incomplete response (SIR) (8–11). In addition, we noticed that most previous studies drew their conclusions just based on final follow-up rather than dynamic follow-ups.

In this study, we tried to investigate the relationship between the timing of initiating RAT and the clinical outcomes based on dynamic follow-ups and assessments in intermediate- to high-risk DTC patients.

## Materials and Methods

The Ethics Committee approved the research of the Tianjin Medical University General Hospital. Written informed consent was given by all patients participating in the study. All clinical data used in this research were anonymized for analysis.

### Patients

**Figure 1:**
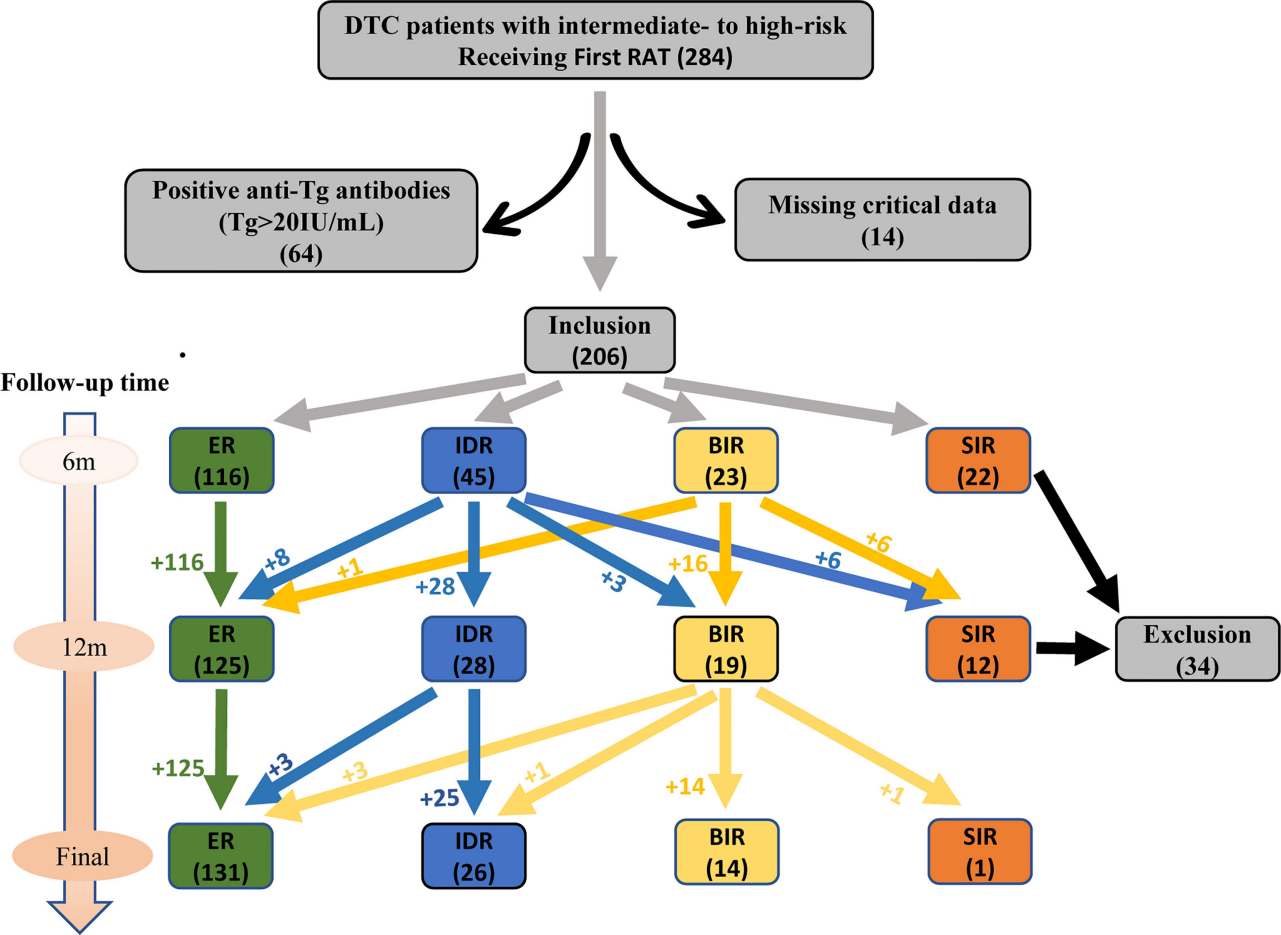
Flow chart for selection process of eligible participants.

We retrospectively collected the clinical records of patients with DTC treated in our department (Department of Nuclear Medicine, Tianjin Medical University General Hospital, Tianjin, China) from October 2016 to March 2021. All these DTC patients underwent total thyroidectomy and received the first post‐thyroidectomy RAT. Patients with intermediate‐ to high‐risk and received RAT dose of 150 mCi were included according to the ATA guidelines (version 2015), while individuals had poor clinicopathologic features that mainly contained anyone of the following characteristics: (i) with T4 status of tumor in situ, (ii) with soft tissue invasion, (iii) with cervical lymph node metastasis of 3 cm or more in diameter and (or) extranodal invasion, (iv) with serum sTg >10 ng/mL. Patients were excluded with anyone of the followings: (i) with tumor of incomplete resection, (ii) with known distant metastases, (iii) post-operative serum Tg level highly suggestive of distant metastases, (iv) positive anti-Tg antibodies (TgAb) (>20 IU/mL, reference value: 0-40 IU/mL), (v) missing critical data. Finally, 206 eligible DTC patients (139 women and 67 men, ratio 2.1:1) were enrolled in this study. The median age was 48 for women and 46 for wen. The flow chart was showed in **Figure 1**.

### Collection of Clinical Data

The patients’ clinical records including gender, age, date of surgery and initial RAT, TSH, sTg, TgAb before RAT and during the dynamic follow-ups, were thoroughly reviewed. The size and status of in-situ tumors and metastatic lymph nodes were obtained from operative recordings and postoperative pathological reports, and then, patients were staged according to the American Joint Cancer Committee (AJCC)/Union for International Cancer Control (UICC) TNM staging system (8th edition). The recurrence risk of individuals was classified based on the ATA guidelines (version 2015).

### Procedures of RAT and Follow-up Protocol

Subsequent RAT was conducted after a low-iodine diet for at least 2 weeks and thyroid hormone withdrawal until the TSH exceeded 30 μIU/mL. During hospitalization, cervical ultrasound and chest computed tomography (CT) would be examined routinely. Whole‐body RAI scans (WBSs) were performed 3 days after ^131^I administration. Afterwards, patients took levothyroxine for thyrotropin suppression routinely. RAI therapy was re-conducted 6 months after RAT in patients if ^131^I-avid persistent/recurrence/metastatic DTC lesions were identified by post WBS.

Patients who had no overt structural/functional disease were followed up for more than one year, meanwhile, they must take levothyroxine regularly for TSH suppression therapy. The follow-up visit and possible modulation of levothyroxine dosage were conducted at 1-, 3-, 6- and 12-months post RAT except as otherwise indicated, then the interval was extended to 6-12 months. Patients were regularly followed up by physical examination and measurements of thyroid hormones, TSH, Tg and TgAb. Neck ultrasound was also conducted during follow-up duration. Additional imaging studies (e.g., WBS, CT) were performed, as needed, whenever the clinical or laboratory findings raised the suspicion of persistent or recurrent disease.

### Study Design and Response Classifications

All patients received RAT after total thyroidectomy. 6m, 12m after RAT and recently, we followed up these patients and evaluated the therapy response. According to the therapy stratification system, the therapy responses to RAT were assessed as either ER, IDR, BIR, or SIR at every follow-up. In addition, BIR and SIR were collectively referred to as incomplete response (IR).

Patients were categorized by their time interval (TI: between thyroidectomy and initial RAT) and divided into 2 groups: Group 1: TI < 3 months (n=148), and Group 2: TI ≥ 3 months (n=58). Then, patients were further categorized by their clinicopathologic features and sTg level and divided into 3 sub-groups: poor clinicopathologic features (PCPF): DTC with poor clinicopathologic features and any sTg level, hyperthyroglobulinemia (hyper-Tg): DTC with sTg >10 ng/mL and no poor clinicopathologic features, PCPF & hyper-Tg: DTC with both poor clinicopathologic features and sTg >10 ng/mL.

The therapy responses were evaluated between the two groups and the three sub-groups in dynamic follow-ups. Notably, the patients would be excluded from the later comparison of the responses who were evaluated as SIR, because these patients received consequent RAI therapy or other therapy. Then, the univariate and multivariate analyses were performed to explore the influence of some factors on patients’ IR rate, such as age, gender, histological type, soft tissue invasion, T and N status, pre-therapy TSH, sTg, and recurrence risk. Finally, the nomogram was developed to explain IR rates for patients as a valuable tool in clinical practice.

### Statistical Analysis

The Mann-Whitney U test was used to compare differences in continuous variables, such as age, Pre-therapy TSH, sTg level, and follow-up duration. A chi-squared test was used to analysis differences in categorical variables, such as gender, histological type, T status, N status, AJCC stage, recurrence risk, soft tissue invasion, and structural or functional Disease existence (SoFD) in the first post-therapy of ^131^I imaging. The univariate analysis was conducted by log-rank test. All *P* values presented were 2-tailed, and values <0.05 were considered to be statistically significant. Variables with statistically significant *P* value in the univariate analysis were included in the Cox regression for independent risk analysis. The establishment of the nomogram was performed using R software (version 4.0.5; http://www.r-project.org/). All statistical analyses were performed using SPSS software version 25.0 (SPSS Inc., Chicago, IL, USA).

## Results

### Patients’ Baseline Characteristics

**Table 1** showed the clinical and pathologic characteristics of group 1 (n=148) and group 2 (n=58). There were no significant differences in age, gender, histological type, T status, N status, AJCC stage, recurrence risk, soft tissue invasion, Pre-therapy TSH, sTg level, SoFD, and follow-up duration between the two groups.

**Table 1:** Clinical and pathologic characteristics.

Characteristics	TI < 3 months (n = 148)	TI ≥ 3 months (n = 58)	P
Age at diagnosis	46 (33-55)	46 (35-55)	0.825^a^
Gender			
Male	44 (29.7)	23 (39.7)	0.171^b^
Female	104 (70.3)	35 (60.3)	
Histological type			
Papillary	145 (98.0)	57 (98.3)	1.000^c^
Follicular	3 (2.0)	1 (1.7)	
T status			
T1	51 (34.5)	15 (25.9)	0.494 ^b^
T2	11 (7.4)	5 (8.6)	
T3	35 (23.6)	12 (20.7)	
T4	51 (34.5)	26 (44.8)	
N status			
N0	14 (9.5)	3 (5.2)	0.085 ^b^
N1a	77 (52.0)	23 (39.7)	
N1b	57 (38.5)	32 (55.2)	
AJCC stage			
I	110 (74.3)	45 (77.6)	0.696 ^b^
II	19 (12.8)	8 (13.8)	
III	19 ( (12.8)	5 (8.6)	
Recurrence Risk			
Intermediate	94 (63.5)	29 (50.0)	0.075^b^
High	54 (36.5)	29 (50.0)	
Soft tissue invasion			
No	77 (52.0)	26 (44.8)	0.353^b^
Yes	71 (48.0)	32 (55.2)	
Pre-therapy TSH (μIU/mL)	68.89 (50.52-95.63)	69.40 (50.62-96.74)	0.994 ^a^
Stimulated Tg (ng/mL)	11.20 (2.65-20.70)	11.20 (2.84-20.70)	0.415 ^a^
SoFD			
Yes	18 (12.2%)	9 (15.5%)	0.830 ^a^
No	130 (87.8%)	49 (84.5%)	
Follow-up duration (months)	22.1 (10.23-23.43)	22.8 (17.23-28.53)	0.400 ^a^

Data are expressed as the median (percentiles 25-75) or frequencies.

SoFD means Structural or Functional Disease existence in the first post-therapy of ^131^I imaging.

**^a^** means Mann-Whitney U test.

**^b^** means Pearson Chi-squared test.

**^c^** means Chi-squared test (continuity correction).

### Comparison of Responses to Initial RAT Between 2 Groups During Dynamic Follow-ups

As showed in **Table 2**, the proportions of ER, IDR, BIR, SIR, Non-ER, IR, Non-IR were statistically different between the two groups (all *P*<0.05). With the extension of the follow-up period, the ER rates of both groups gradually increased, on the contrary, the IR rates gradually decreased. Besides, group 2 had significantly lower ER rates (37.9 *vs* 63.5, 52.0 *vs* 73.9, 64.4 *vs* 80.3, all *P*<0.05, respectively) and higher IR rates (39.7 *vs* 14.9, 36.0 *vs* 9.7, 12.2 *vs* 3.9, all *P*<0.05, respectively) than group 1 during dynamic follow-ups. But apart from that, the **Table 2** showed it had no significant difference in the proportions of SIR, Non-SIR between the two groups.

**Table 2:** Comparison of response to initial RAT between 2 groups [n (%)].

Response	6m follow-up		P	12m follow-up		P	Final follow-up		P
	TI < 3 m (n= 148)	TI≥3 m (n = 58)		TI < 3 m (n= 134)	TI≥3 m (n = 50)		TI < 3 m (n =127)	TI≥3 m (n = 45)	
ER	94 (63.5)	22 (37.9)	**0.001^b^**	99 (73.9)	26 (52.0)	**0.000^d^**	102 (80.3)	29 (64.4)	NA
IDR	32 (21.6)	13 (22.4)		22 (16.4)	6 (12.0)		20 (15.7)	6 (13.3)	
BIR	8 (5.4)	15 (25.9)		6 (4.5)	13 (26.0)		4 (3.1)	10 (22.2)	
SIR	14 (9.5)	8 (13.8)		7 (5.2)	5 (10.0)		1 (0.8)	0 (0)	
ER	94 (63.5)	22 (37.9)	**0.001^b^**	99 (73.9)	26 (52.0)	**0.005^b^**	102 (80.3)	29 (64.4)	**0.032^b^**
Non-ER	54 (36.5)	36 (62.1)		35 (26.1)	24 (48.0)		25 (19.7)	16 (35.6)	
IR	22 (14.9)	23 (39.7)	**0.000^b^**	13 (9.7)	18 (36.0)	**0.000^b^**	5 (3.9)	10 (12.2)	**0.001^c^**
Non-IR	126 (85.1)	35 (60.3)		121 (90.3)	32 (64.0)		122 (96.1)	35 (77.8)	
SIR	14 (9.5)	8 (13.8)	0.452**^b^**	7 (5.2)	5 (10.0)	0.406 **^c^**	1 (0.8)	0 (0)	**NA**
Non-SIR	134 (90.5)	50 (86.2)		127 (94.8)	45 (90.0)		126 (99.2)	45 (100.0)	


**^b^** means Pearson Chi-squared test.

**^c^** means Chi-squared test (continuity correction).

**^d^** means Chi-squared test (fisher’s exact).

NA means not applicable.

### Comparison of Responses to Initial RAT Between 2 Groups of Patients in Different Sub-Groups

As showed in **Table 3**, all proportions of ER, IDR, BIR, SIR, Non-ER, IR, Non-IR in the 3 sub-groups respectively were statistically different between the two groups (all *P*<0.05). With the extension of the follow-up period, the ER rates of both groups in the 3 sub-groups gradually increased, on the other hand, the IR rates gradually decreased. Besides, the sub-group PCPF showed that group 2 had significantly lower ER rates (36.2 *vs* 66.3, 51.2 *vs* 73.4, 62.2 *vs* 79.5, all *P*<0.05, respectively) and higher IR rates (42.6 *vs* 12.9, 39.0 *vs* 10.6, 24.3 *vs* 4.5, all *P*<0.05, respectively) than group 1 at the 3 different follow-up time points. Similarly in the left two sub-groups (with hyper-Tg or PCPF & hyper-Tg), group 2 had significantly lower ER rates and higher IR rates during dynamic follow-ups (all *P*<0.05). Additionally, there were no significant difference in the proportions of SIR, Non-SIR between the two groups in the sub-group PCPF/hyper-Tg/PCPF & hyper-Tg.

**Table 3:** Comparison of responses to initial RAT between 2 groups in sub-groups [n (%)].

Response	6m follow-up		P	12m follow-up		P	Final follow-up		P
	TI < 3 m	TI≥3 m		TI < 3 m	TI≥3 m		TI < 3 m	TI≥3 m	
PCPF	n=101	n = 47		n= 94	n = 41		n = 88	n = 37	
ER	67 (66.3)	17 (36.2)	**0.000^c^**	69 (73.4)	21 (51.2)	**0.000^d^**	70 (79.5)	23 (62.2)	NA
IDR	21 (20.8)	10 (21.3)		15 (16.0)	4 (9.8)		14 (15.9)	5 (13.5)	
BIR	6 (5.9)	14 (29.8)		4 (4.3)	12 (29.3)		3 (3.4)	9 (24.3)	
SIR	7 (6.9)	6 (12.8)		6 (6.4)	4 (9.8)		1 (1.1)	0 (0)	
ER	67 (66.3)	17 (36.2)	**0.001^b^**	69 (73.4)	21 (51.2)	**0.012^b^**	70 (79.5)	23 (62.2)	**0.042^b^**
Non-ER	34 (33.7)	30 (63.8)		25 (26.6)	20 (48.8)		18 (20.5)	14 (37.8)	
IR	13 (12.9)	20 (42.6)	**0.000^b^**	10 (10.6)	16 (39.0)	**0.000^b^**	4 (4.5)	9 (24.3)	**0.003^c^**
Non-IR	88 (87.1)	27 (57.4)		84 (89.4)	25 (61.0)		84 (95.5)	28 (75.7)	
SIR	7 (6.9)	6 (12.8)	0.392^b^	6 (6.4)	4 (9.8)	0.741^c^	1 (1.1)	0 (0)	NA
Non-SIR	94 (93.1)	41 (87.2)		88 (93.6)	37 (90.2)		87 (98.9)	37 (100.0)	
**Hyper-Tg**	**n= 80**	**n = 31**		**n= 67**	**n = 24**		**n = 63**	**n = 20**	
ER	42 (52.5)	6 (19.4)	**0.001^b^**	46 (68.7)	7 (29.2)	**0.000^d^**	48 (76.2)	9 (45.0)	NA
IDR	18 (22.5)	7 (22.6)		12 (17.9)	4 (16.7)		11 (17.5)	3 (15.0)	
BIR	7 (8.8)	11 (35.5)		5 (7.5)	9 (37.5)		3 (4.8)	8 (40.0)	
SIR	13 (16.3)	7 (22.6)		4 (6.0)	4 (16.7)		1 (1.6)	0 (0.0)	
ER	42 (52.5)	6 (19.4)	**0.002^b^**	46 (68.7)	7 (29.2)	**0.001^b^**	48 (76.2)	9 (45.0)	**0.009^b^**
Non-ER	38 (47.5)	25 (80.6)		21 (31.3)	17 (70.8)		15 (23.8)	11 (55.0)	
IR	20 (25.0)	18 (58.1)	**0.001^b^**	9 (13.4)	13 (54.2)	**0.000^c^**	4 (6.3)	8 (40.0)	**0.001^c^**
Non-IR	60 (75.0)	13 (41.9)		58 (86.6)	11 (45.8)		59 (93.7)	12 (60.0)	
SIR	13 (16.3)	7 (22.6)	0.436^b^	4 (6.0)	4 (16.7)	0.243^c^	1 (1.6)	0 (0.0)	NA
Non-SIR	67 (83.8)	24 (77.4)		63	20		62 (98.4)	20 (100.0)	
**PCPF&Hyper-Tg**	**n= 33**	**n = 20**		**n= 27**	**n = 15**		**n = 24**	**n = 12**	
ER	15 (45.5)	1 (5.0)	**0.004^d^**	16 (59.3)	2 (13.3)	**0.005^d^**	16 (66.7)	3 (25.0)	NA
IDR	7 (21.2)	4 (20.0)		5 (18.5)	2 (13.3)		5 (20.8)	2 (16.7)	
BIR	5 (15.2)	10 (50.0)		3 (11.1)	8 (53.3)		2 (8.3)	7 (58.3)	
SIR	6 (18.2)	5 (25.0)		3 (11.1)	3 (20.0)		1 (4.2)	0 (0.0)	
ER	15 (45.5)	1 (5.0)	**0.002^b^**	16 (59.3)	2 (13.3)	**0.004^b^**	16 (66.7)	3 (25.0))	**0.018^b^**
Non-ER	18 (54.5)	19 (95.0)		11 (40.7)	13 (86.7)		8 (33.3)	9 (75.0)	
IR	11 (33.3)	15 (75.0)	**0.003^b^**	6 (22.2)	11 (73.3)	**0.001^b^**	3 (12.5)	7 (58.3)	**0.012^c^**
Non-IR	22 (66.79)	5 (25.0)		21 (77.8)	4 (26.7)		21 (87.5)	5 (41.7)	
SIR	6 (18.2)	5 (25.0)	0.807^c^	3 (11.1)	3 (20.0)	0.649^d^	1 (4.2)	0 (0.0)	NA
Non-SIR	27 (81.8)	15 (75.0)		24	12		23 (95.8)	12 (100.0)	

**^b^** means Pearson Chi-squared test.

**^c^** means Chi-squared test (continuity correction).

PCPF means poor clinicopathologic features; Hyper-Tg means hyperthyroglobulinemia; PCPF&Hyper-Tg means both PCPF and Hyper-Tg.

### Univariate and Multivariate Cox Analysis for Potential Risk Factors of IR to RAT

**Figure 2:**
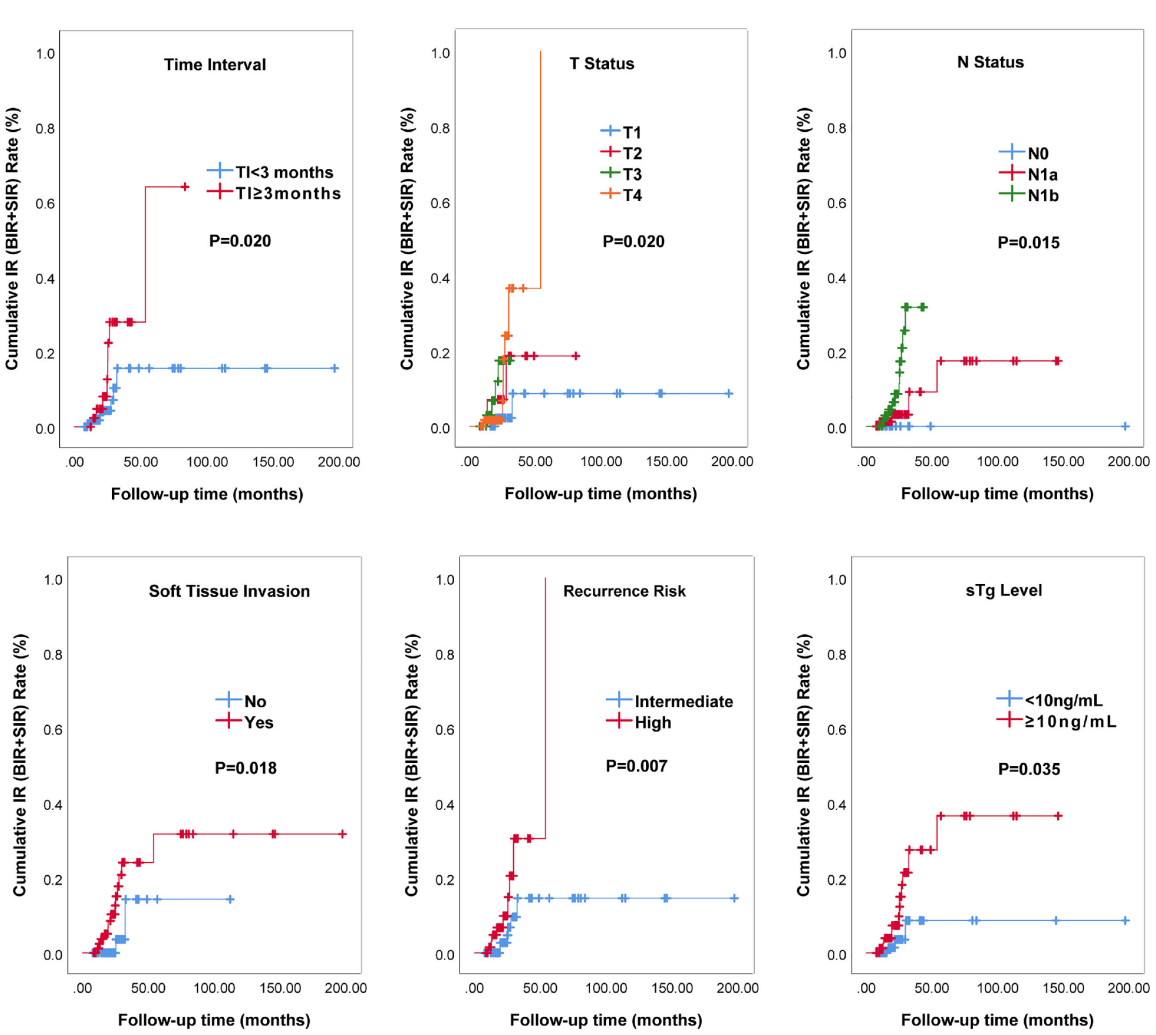
IR curves of intermediate‐ to high‐risk DTC patients receiving RAT analyzed by Kaplan-Meier test.

**Figure 2** and **Table 4** showed the results of univariate Cox analysis about risk factors of IR to RAT. According to the univariate analysis, 6 significant variates including TI, T status, N status, soft tissue invasion, recurrence risk, and sTg level were risk factors for IR (all *P*<0.05), while there was no significant difference in other variables (all *P*>0.05). To further explore the predictive value, we entered these 6 risk factors into a multivariate Cox regression analysis model. The result showed that prolonged TI (HR: 6.67, 95%CI: 2.241-19.857, *P*=0.001), soft tissue invasion (HR: 7.35, 95%CI: 1.624-33.296, *P*=0.010), higher sTg (HR: 7.21, 95%CI: 1.991-26.075, *P*=0.003) were manifested to be independent risk factors for IR.

**Table 4:** Univariate and multivariate Cox regression analysis based on all variables for IR.

Characteristics	Univariate analysis		Multivariate analysis	
	HR (95%CI)	P	HR (95%CI)	P
Age at diagnosis				
≤45	Reference			
>45	1.376 (0.489-3.868)	0.546		
Gender				
Male	Reference			
Female	1.197 (0.424-3.377)	0.734		
Histological type				
Papillary	Reference			
Follicular	21.187(0.000-10^7)	0.655		
T status				
T1	Reference			
T2	3.996 (0.551-28.981)	0.171		
T3	11.237 (1.810-69.755)	**0.009**		
T4	8.133 (1.584-41.774)	**0.012**		
N status				
N0	Reference			
N1a	1.436 (0.763-27.562)	0.145		
N1b	2.436 (1.768-34.682)	**0.036**		
AJCC stage				
I	Reference			
II	0.380 (0.049-2.961)	0.356		
III	1.813 (0.394-8.334)	0.445		
Recurrence Risk				
Intermediate	Reference			
High	3.918 (1.354-11.337)	**0.012**		
Soft tissue invasion				
No	Reference		Reference	
Yes	5.080 (1.139-22.664)	**0.033**	7.353 (1.624-33.296)	**0.010**
Pre-therapy TSH (μIU/mL)				
30-60	Reference			
>60	1.184 (0.708-1.980)	0.520		
Stimulated Tg (ng/mL)				
<10	Reference		Reference	
≥10	3.587 (1.009-12.754)	**0.048**	7.205 (1.991-26.057)	**0.010**
SoFD				
Yes	Reference			
No	1.562 (0.352-6.933)	0.558		
Time interval (month)				
<3	Reference		Reference	
≥3	5.623 (1.913-16.527)	**0.002**	6.671 (2.241-19.957)	**0.001**

### Prognostic Nomogram for IR Rates to RAT

**Figure 3:**
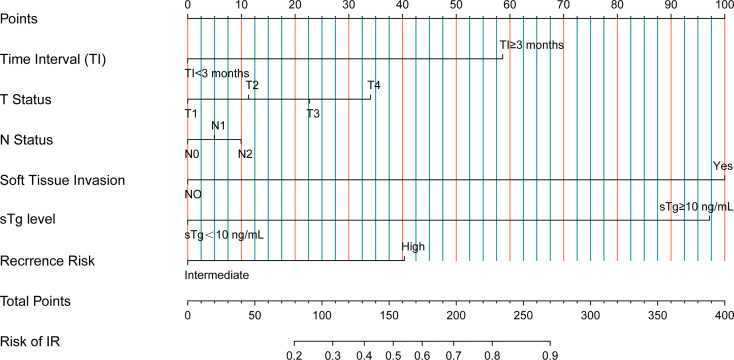
Nomogram predicting IR rates for intermediate‐ to high‐risk DTC patients receiving RAT.

Based on the risk factors from univariate Cox analysis, we constructed a nomogram for predicting the IR rate. Each variable was assigned a score on a scale. By adding scores for each of the selected variables, a total score was obtained. Then a vertical line was dropped down from the total points row to estimate the risk of IR. The nomogram showed that soft tissue invasion, sTg, and TI were the top 3 contributors to the IR, followed by recurrence risk, T status, and N status (**Figure 3**).

## Discussion

RAI therapy for DTC has been used for a number of years, but the relationships between RAI initiating time and clinical outcomes are still not explicit. Recently, several studies have noted on this tissue with controversial conclusions. Scheffel et al. showed that the time (≥6 months after thyroidectomy) between total thyroidectomy and RAI therapy does not interfere with the response to initial therapy, disease status at follow‐up and recurrence rate in patients with DTC (8). Similarly, Kim et al (7) demonstrated that delaying the first RAI therapy until 6 months after total thyroidectomy had no impact on restaging, recurrence and mortality in intermediate‐ to high‐risk DTC. However, Li et al (3) reported that delayed initial RRA (≥3 months after thyroidectomy) related to IR in low- to intermediate-risk DTC. However, the current DTC guidelines have no recommendations about the timing of RAI therapy (6, 12, 13). Accordingly, concerns about the best timing for RAI administration are shared among patients and physicians in clinical practice.

This study evaluated the impact of timing of the RAI therapy in intermediate‐ to high‐risk patients with clinically characterized DTC. Notably, our study had something different from other studies. Firstly, the patients enrolled in our study all received first post‐thyroidectomy RAT of potential residual tumor, neither RRA of remnant thyroid gland nor RTKD. Secondly, the therapy response was based on dynamic follow-ups and assessments, not single checking-time points. Therefore, our findings provide evidence‐based guidance on the appropriate timing of initial RAT administration in managing intermediate‐ and high‐risk patients with DTC.

We have evaluated the impact of the timing of RAT on intermediate‐ to high‐risk DTC patients’ outcomes followed by dynamic follow-ups at 6m, 12m, and the last time after the first RAT. We showed that delayed initial RAT (≥3 months after thyroidectomy) may cause higher IR rates in intermediate- to high-risk DTC patients during dynamic follow-ups. Our findings were contrast with those reported by Kim et al. and Scheffel et al, which significant methodological differences may partially explained. The therapy response evaluation methods used in these two studies mainly based on disease free survival (DFS) and overall survival (OS). However, OS is insufficient and inappropriate to assess the clinical outcomes of all DTC patients, especially for those with a wonderful 5-year OS of more than 95% (14). Differently, we used the four-tiered therapy response evaluation system proposed by ATA guideline (version 2015), including ER, IDR, BIR and SIR. This new therapy response evaluation system confirmed that it could effectively predict the risk of recurrence and persistent disease, thereby providing dynamic risk estimates of responses to prior RAI therapy that can be used to tailor ongoing follow-up recommendations (15).

In our center, the DTC patients immediately took levothyroxine for thyrotropin suppression after thyroidectomy. One month after that, serum Tg, TgAb, thyroid hormones and neck ultrasound were measured and used to evaluate patients’ status. Then, according to the patients’ disease status and recurrence risk stratification, an individualized therapy strategy would be made for every patient. If necessary, subsequent RAT was conducted after a low-iodine diet for at least 2 weeks and thyroid hormone withdrawal until the TSH exceeded 30 μIU/mL. Generally, most patients (accounts for about 71.8% of DTC patients during the study) completed their first RAT within three months after thyroidectomy, which may better reflect current practice patterns in the care of patients with DTC.

An earlier Meta-Analysis detailed that poor clinicopathologic features may relate to the occurrence of radioiodine refractory DTC (16). Tg reduction index and sTg level may be useful in predicting clinical outcomes and potential future disease recurrence in DTC (17–19). To further analyze the impact of poor clinicopathologic features and sTg level on response to initial RAT within two groups, we set three sup-groups based on clinicopathologic features and sTg level. The results showed that delayed initial RAT indicated higher IR rates during dynamic follow-ups, which was consistent with the general comparison of response to initial RAT between 2 groups.

Further univariate and multivariate analyses confirmed the TI (≥3 months) to be an independent risk factor for IR. The IR rate of patients in the delayed group was 6.67 times higher than those who received RAT within three months. During the prolonged interval, the potential progression of microlesions may exist for a possible explanation of this observation, which was the possible mechanism in recurrence and distant metastatic DTC (3, 20). Furthermore, two other independent risk factors for IR, including soft tissue invasion and sTg level, were selected in multivariate Cox regression analysis. These findings were similar to Yang et al (21), who illustrated that extrathyroidal invasion was an important predictive factor (P=0.05) for SIR, and higher preablative sTg and tumor size were independent predictive factors for SIR (P<0.001, P=0.002, respectively). Additionally, different from Li et al (3), we demonstrated a significant relationship between soft tissue invasion/higher sTg level and IR. Different research populations might be responsible for these different results, as we only focused on intermediate- to high-risk DTC, while they only focused on low- to intermediate-risk DTC. Therefore, we suggested that prolonged TI, soft tissue invasion and higher sTg level might predict higher IR rates in patients with intermediate‐ to high‐risk DTC receiving RAT. The patients with any of above three independent risk factors should need additional evaluations and possibly even complementary therapies.

In addition, to better determine the prognosis in intermediate- to high-risk DTC patients, we constructed a nomogram to predict IR rates based on 6 risk factors, including TI, T status, N status, soft tissue invasion, sTg level, and recurrence risk. From the nomogram, we could intuitively see that soft tissue invasion, sTg level, and TI were the top 3 contributors to the IR, which were consistent with the results of multivariate Cox analysis.

Some limitations in our study must be considered. Firstly, the sample size was small. Secondly, the follow-up period was short and only RAI therapy responses were analyzed, but not DFS and recurrence rates. Thirdly, the exclusion of patients with SIR could cause data bias and the simple Chi-Square test may not be the most appropriate statistical approach when analyzing time dependent change. Finally, only a few patients (21/206) with TI more than 6 months were enrolled in our study, resulting in the impact of longer TI on IR cannot be assessed.

## Conclusions

In conclusion, early RAT is associated with greater biochemical response but has no impact on SIR. Our study showed that prolonged TI (≥3 months), Soft tissue invasion, and higher sTg level might predict higher IR rates in patients with intermediate‐ to high‐risk DTC receiving RAT. These patients may benefit from timely RAI therapy. The nomogram developed in this study may be a valuable tool when explaining IR rates to patients in clinical practice.

## Data Availability Statement

The raw data supporting the conclusions of this article will be made available by the authors, without undue reservation.

## Ethics Statement

The studies involving human participants were reviewed and approved by The Ethical Committee of Tianjin Medical University General Hospital. The patients/participants provided their written informed consent to participate in this study.

## Author Contributions

FY and XL share first authorship. All authors contributed to the article and approved the submitted version.

## Conflict of Interest

The authors declare that the research was conducted in the absence of any commercial or financial relationships that could be construed as a potential conflict of interest.

## Publisher’s Note

All claims expressed in this article are solely those of the authors and do not necessarily represent those of their affiliated organizations, or those of the publisher, the editors and the reviewers. Any product that may be evaluated in this article, or claim that may be made by its manufacturer, is not guaranteed or endorsed by the publisher.
